# Supplementary material for: Unravelling the origins of ice nucleation on organic crystals
Source: Chem Sci. 2018 Aug 27;9(42):8077–88. doi: 10.1039/c8sc02753f (PMC6238755; doi:10.1039/c8sc02753f)
Supplement: Supplementary file 1 [file SC-009-C8SC02753F-s001.pdf]

Electronic Supplementary Material (ESI) for Chemical Science.
This journal is © The Royal Society of Chemistry 2018

Unravelling the Origins of Ice Nucleation on Organic Crystals: SUPPLEMENTARY INFORMATION

Gabriele C. Sosso,* Thomas F. Whale, Mark Holden, Philipp Pedevilla, Benjamin J. Murray, and Angelos Michaelides
Department of Chemistry and Centre for Scientific Computing, University of Warwick, Gibbet Hill Road, Coventry CV4 7AL, United Kingdom
Thomas Young Centre, London Centre for Nanotechnology and Department of Physics and Astronomy, University College London, London WC1E 6BT, United Kingdom
School of Earth and Environment and School of Chemistry, University of Leeds, Leeds LS2 9JT, United Kingdom and
Institute for Climate and Atmospheric Science, School of Earth and Environment, University of Leeds, Leeds LS2 9JT, United Kingdom

We provide supplementary material about our atomistic simulation of the water-cholesterol monohydrate (CHLM) interface. We discuss:

- The reliability of the force fields we have used
- The details of our forward flux sampling simulations
- The characterisation of the morphology of the ice nuclei
- The role of the flexibility of the CHLM surface
- The ice nucleating ability of anhydrous cholesterol (CHLA)

VALIDATION OF THE FORCE FIELDS

A major concern when it comes to atomistic simulations of water at complex interface, is whether or not a given combination of force fields can accurately reproduce the interaction between, in this case, water molecules and cholesterol crystals. This is especially important in the context of simulations of ice formation, as even small structural details have the potential to significantly alter the kinetics of nucleation [1].

Here we present a validation of our computational setup (CHARMM36 [2, 3] for the cholesterol and TIP4P/Ice [4] for water). As a start, we note that even if the CHARMM36 has been originally parametrized to be used in conjunction with the TIP3P water model [5], a number of recent works (see e.g. Refs. 6, 7 verified the accuracy of the CHARMM36/TIP4P combination.

As it concerns the reliability of CHARMM36 alone in reproducing the structure of CHLM crystals, we report in Table I the cell parameters (cell vectors and angles) of bulk cholesterol monohydrate at zero K. Experimental (Exp., Ref. 8) values are compared with Density Functional Theory calculations employing dispersion inclusive GGA exchange-correlation functionals: vdW-DF, as reported in Ref. 9, vdW-DF (this work), OPTB88_VDW (Exp.) (starting from the experimental configuration) and OPTB88_VDW (min[CHARMM36]) (starting from the equilibrium configuration as calculated using the CHARMM36 force field). The results obtained using the CHARMM36 force field are also reported (CHARMM36). The small mismatch with respect to both the experimental and the ab initio data (of the order of few %) confirms the reliability of CHARMM36 in describing the CHLM structure we have considered in this work.

Concerning the computational details of DFT calculations, they were performed using the mixed Gaussian and Plane-Waves (GPW) method implemented in the CP2K package [10]. We used either the vdW-DF [11] and the optB88-vdW [12] exchange-correlation functional and Goedecker-type pseudopotentials [13] with four, one and six valence electrons for C, H, and O respectively. The Kohn-Sham (KS) orbitals were expanded in a Double-ZetaValence plus Polarization (DZVP) Gaussian-type basis set, while the plane wave cutoff for the finest level of the multi-grid [13] has been set to 400 Ry to efficiently solve the Poisson equation within periodic boundary conditions using the Quickstep scheme [10]. Brillouin zone integration was restricted to the supercell Γ-point. 2x2x2 and 3x3x1 supercells have been considered for bulk and slab geometries respectively. In the latter case, a vacuum region of ~30 Å has been inserted between the periodic replica of the slabs along the [001] direction normal to the cleavage plane. These settings ensure an accuracy of the resulting total energy of 4 meV/atom. When necessary, a dipole correction has been applied to account for the non-uniform charge distribution across e.g. a single slab of CHLM.

In Fig. S1a and Fig. S1b we report the binding energy curves for a single water molecule interacting with the hydroxyl group of a single cholesterol molecule. The result obtained via the TIP4P/Ice-CHARMM36 force fields (CHARMM) are compared with Density Functional Theory calculations using either the VDW-DF or the OPTB88-VDW exchange correlation functionals. The binding energies have been calculated across the paths

* G.Sosso@warwick.ac.uk

Bulk @ 0 K

Cholesterol Monohydrate						
	a [Å]	b [Å]	c [Å]	α [deg]	β [deg]	γ [deg]
Exp. [8]	12.39	12.41	34.36	91.9	98.1	100.8
vdW-DF [9]	12.37	12.47	34.50	92.6	98.8	101.7
vdW-DF	12.39	12.53	34.46	92.7	98.4	101.8
OPTB88_VDW (Exp.)	12.01	12.09	33.88	93.2	99.3	102.6
OPTB88_VDW (min[CHARMM36])	11.75	12.34	33.79	93.5	97.5	100.5
CHARMM36	11.94	12.43	33.81	93.2	96.6	100.2

TABLE I: Cell parameters a, b and c, and cell angles α, β and γ of bulk cholesterol monohydrate at zero K. Experimental (Exp., Ref. 8) values are compared with Density Functional Theory calculations employing dispersion inclusive GGA exchange-correlation functionals: vdW-DF, as reported in Ref. 9, vdW-DF (this work), OPTB88_VDW (Exp.) (starting from the experimental configuration) and OPTB88_VDW (min[CHARMM36]) (starting from the equilibrium configuration as calculated using the CHARMM36 force field). The results obtained using the CHARMM36 force field are also reported (CHARMM36).

shown in the insets of Fig. S1, that is as a function of either the distance between the hydrogen of the CHL hydroxyl group and the oxygen of the water molecule or the distance between the oxygen of the CHL hydroxyl group and one of the hydrogens of the water molecule. The mismatch between TIP4P/Ice-CHARMM36 and DFT is comparable to that we observe between the two different exchange-correlation functional. Furthermore, we show in Fig. S1c a scatter plot (DFT OPTB88_VDW versus CHARMM36) of the binding energies for selected configurations of water on a CHLM slab. S_{CH3} and S_{OH} refers to CHLM monolayers exposing the hydrophobic -CH3 terminated or the hydrophilic -OH terminated face to the water respectively. Different combinations of the interactions between of O_{H2O} (oxygen of the water molecule), H_{H2O} (one of the hydrogens of the water molecule), O_{H2O} (oxygen of the -OH group), H_{OH} (hydrogen of the -OH group) and H_{CH3} (hydrogen of the -CH3 group) have been considered. The red line and the surrounding shaded region correspond to the ideal correlation between DFT OPTB88_VDW and CHARMM36 energies, comprehensive of the uncertainty originating from the different possible orientations (which have been probed at the CHARMM36 level) corresponding to the various combinations described above. Overall the resulting energy ranking is very well reproduced by the TIP4P/Ice-CHARMM36 setup, and as a whole we are thus confident that our computational framework is adequate to perform simulations of heterogeneous ice nucleation for this particular system.

FORWARD FLUX SAMPLING SIMULATIONS

Order Parameter

The first step in setting up the FFS simulation involved choosing a suitable order parameter λ. We start by labelling as ice-like any water molecule whose oxygen atom displays a value of lq^6¿0.45, where lq^6 is constructed as follows: we first select only those oxygens which are hydrogen-bonded to four other oxygens. For each of the $i-$th atoms of this subset S_{4HB}, we calculate the local order parameter:

$$lq_i^6 = \frac{\sum_{j=1}^{N_{S_{4HB}}} \sigma(\mathbf{r}_{ij}) \sum_{m=-6}^{6} q_{i,m}^{6*} \cdot q_{j,m}^6}{\sum_{j=1}^{N_{S_{4HB}}} \sigma(\mathbf{r}_{ij})} \quad (1)$$

where $\sigma(\mathbf{r}_{ij})$ is a switching function tuned so that $\sigma(\mathbf{r}_{ij})$=1 when atom j lies within the first coordination shell of atom i and which is zero otherwise. $q_{i,m}^6$ is the Steinhardt vector [14]

$$q_{i,m}^6 = \frac{\sum_{j=1}^{N_{S_{4HB}}} \sigma(\mathbf{r}_{ij}) Y_{6m}(\mathbf{r}_{ij})}{\sum_{j=1}^{N_{S_{4HB}}} \sigma(\mathbf{r}_{ij})}, \quad (2)$$

$Y_{6m}(\mathbf{r}_{ij})$ being one of the 6th order spherical harmonics. We have used 3.2 Å as the cutoff for $\sigma(\mathbf{r}_{ij})$ to be consistent with Ref. 15. Note that by selecting oxygen atoms within the S_{4HB} subset exclusively we ensure that the hydrogen bond network within the ice nuclei is reasonable. Having identified a set of ice-like water molecules, we pinpoint all the connected clusters of oxygen atoms which: i) belong to the S_{4HB} subset; ii) have a value of lq^6¿0.45 and; iii) are separated by a distance $\leq$ 3.2 Å. We then select the largest of these clusters (i.e. the one containing the largest number of oxygen atoms or equivalently water molecules). The final step is to find all the *surface molecules* that are connected to this cluster, as this procedure allows us to account for the diffuse interface between the solid and the liquid. Surface molecules are defined as the water molecules that lie within 3.2 Å from the molecules in the cluster. The final order parameter λ used in this work is thus the number of water molecules within the largest ice-like cluster plus the number of surface molecules. This approach allow us to include ice-like atoms sitting directly on top of the kaolinite surface, which are never labelled as ice-like (and which would thus never be included into the ice nuclei) because

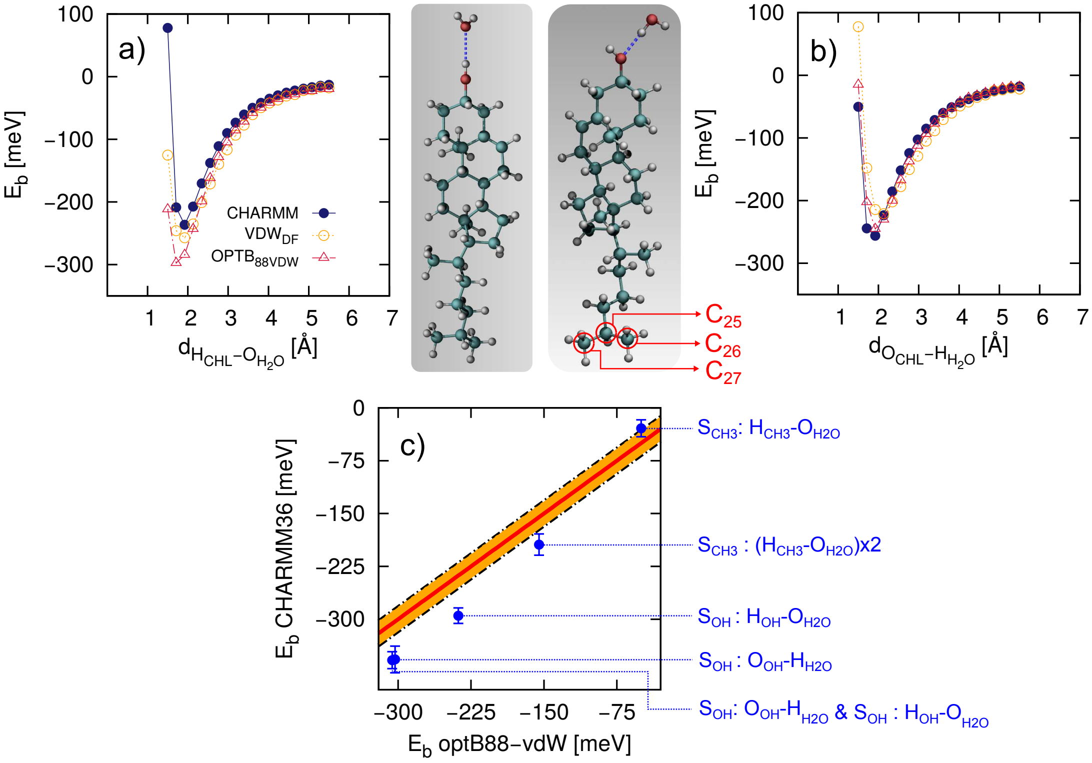

FIG. 1: Binding energy curves for a water monomer hydrogen bonded to the hydroxyl group -OH of a single cholesterol molecule. a) and b) refers to the situation where the hydroxyl group acts as hydrogen bond acceptor and donor respectively, as depicted in the insets. The result obtained via the TIP4P/Ice-CHARMM36 force fields (CHARMM) are compared with Density Functional Theory calculations using either the VDW-DF (VDW_{DF}) or the OPTB88-VDW ($OPTB_{88VDW}$) exchange correlation functionals. The binding energies have been calculated across the paths shown in the insets, that is as a function of either a) the distance between the hydrogen of the CHL hydroxyl group and the oxygen of the water molecule (d H_{CHL}-O_{H_2O}) or b) the distance between the oxygen of the CHL hydroxyl group and one of the hydrogens of the water molecule (d O_{CHL}-H_{H_2O}). c) Scatter plot (DFT OPTB88_VDW versus CHARMM36) of the binding energies for selected configurations of water on a CHLM slab. S_{CH3} and S_{OH} refers to CHLM monolayers exposing the hydrophobic -CH3 terminated or the hydrophilic -OH terminated face to the water respectively. Different combinations of the interactions between of O_{H2O} (oxygen of the water molecule), H_{H2O} (one of the hydrogens of the water molecule), O_{H2O} (oxygen of the -OH group), H_{OH} (hydrogen of the -OH group) and H_{CH3} (hydrogen of the -CH3 group) have been considered. The red line and the surrounding shaded region correspond to the ideal correlation between DFT OPTB88_VDW and CHARMM36 energies, comprehensive of the uncertainty originating from the different possible orientations (which have been probed at the CHARMM36 level) corresponding to the various combinations described above.

they are under coordinated and because they display a different symmetry to the molecules within bulk water (which in turn leads to different values of lq^6). Note that the order parameter used in Ref. 15 differs with respect to our formulation in that i) a slightly stricter criterion has been used to label molecules as ice-like, namely lq^6¿0.5 to be compared with our choice of lq^6¿0.45; and ii) surface molecules are not included in the largest ice-like nucleus. This means that in order to compare quantitatively our results with those of Ref. 15 in terms of e.g. the size of the critical nucleus, the very same order parameter has to be used. The calculation of the order parameter is performed on the fly during our MD simulations thanks to the flexibility of the PLUMED plugin [16, 17] (version 2.2). This code deals chiefly with metadynamics simulations, but can be adapted to a FFS simulation. Note that PLUMED benefits from a fully parallel implementation that flawlessly couples with the GPU-accelerated version of GROMACS, and thus provides a very fast tool for performing FFS simulations. Indeed, while several implementations of FFS are beginning to appear, the main issue preventing wider adoption remains the implementation of the order parameter, which can be as complex as the one used in this work. PLUMED allows a wide range of order parameters to be exploited without the need to re-code them elsewhere.

Converging the Flux Rate and the Individual Crossing Probabilities

In order to calculate the flux rate Φ_0 we have performed a 1.2 ms long unbiased MD simulation, and subsequently built a probability density distribution for $P(\lambda)$. We have thus delimited the liquid basin in terms of the order parameter as $0 < \lambda < \lambda_{Liq} = 24$, while setting the initial interface for the FFS λ_0=65, corresponding to a value of the cumulative distribution function of $P(\lambda)$ of 0.99. The flux rate is then computed as the number of direct crossings of λ_0 (i.e. coming from $\lambda < \lambda_{Liq}$) divided by the total simulation time, and as such should flatten as a function of time. Meanwhile, the number of direct crossings should increase linearly with time. The value obtained for Φ_0 and the number of crossings as a function of time are reported in Fig. S2a. This figure demonstrates that, as previously noted in Refs. 18, 19, long simulation times are needed in order to converge this quantity for inhomogeneous systems. The calculated value of Φ_0 is 0.00094 ps^{-1}, which normalised by the average volume of the water film (60475.56 Å^3) leads to the final value of $1.5{\cdot}10^{-8\pm1}$ ps^{-1} Å^{-3}. Note that we have chosen to normalise the flux rate by the average volume of the water film instead of by the surface area for the slab. While the latter choice could in principle be thought as more meaningful in the context of heterogeneous nucleation, our objective is to compare our numbers with homogeneous ice formation [15] and ice formation on kaolinite [19], which is why we choose the volume normalisation rather than the surface area one. However, it should be noticed that the two different normalisations only introduce a difference of an order of magnitude in the nucleation rate. The number of starting configurations, one for each direct crossing of λ_0, is of the order of 10^3, providing a comprehensive sampling including ice-like clusters in the bulk of the water film as well as on top of the water surface.

Converging the individual crossing probabilities $P(\lambda_i|\lambda_{i-1})$ required in our case as many as 10,000 trial MD runs for the first few interfaces. The initial velocities for each MD run were randomly initialised consistent with the corresponding Maxwell-Boltzmann distribution at 230 K. In line with the coarse graining approach discussed in Ref. 15, we have decided to compute the value of λ on the fly every 4 ps, a frequency far smaller than the relaxation time of the liquid at this temperature (about 0.5 ns) which allows us to neglect meaningless fluctuation on very short timescales. Note that at the interfaces corresponding to critical/post-critical ice nuclei a much smaller number (about 500) of trial MD runs have been shot, as for large ice nuclei to get back to the liquid phase simulation times of the order of 10-40 ns are needed, dramatically increasing the computational cost - albeit more and more nuclei proceed to grow as λ increases leading to a faster convergence of the crossing probabilities. The confidence intervals for each $P(\lambda_i|\lambda_{i-1})$ have been computed according to the binomial distribution of the number of successful trial runs collected at λ_i (see e.g. Ref. [20]).

We report in Fig. S2b the calculated growth probability $P(\lambda|\lambda_0)$ as a function of lambda, together with the fraction of ice nuclei that can be found at the water-CHLM_{001}^{-OH} interface (according to the criterion illustrated in the following "Spatial extent Δz" section). $P(\lambda|\lambda_0)$ converges for λ=350, a value larger than the threshold ($\lambda \sim$200) beyond which finite size effects start to affect significantly our simulations, as discussed in the SI. Nonetheless, it can be clearly seen that the fraction of ice nuclei at the water-CHLM_{001}^{-OH} interface is very large ($\sim$ 75%) from the very beginning, and that for λ=125 no nuclei within the bulk of the water slab survive, indicating a strong preference for ice to form at the water-CHLM_{001}^{-OH} interface. We have observed a similar trend in the case of ice nucleation on the hydroxylated (001) basal face of kaolinite [19], but the fraction of ice nuclei at the water-kaolinite interface at the initial stages of the FFS algorithm was much smaller ($\sim$ 25%). This suggests that pre-critical ice-like fluctuations are much more likely to occur at the surface of CHLM compared to kaolinite.

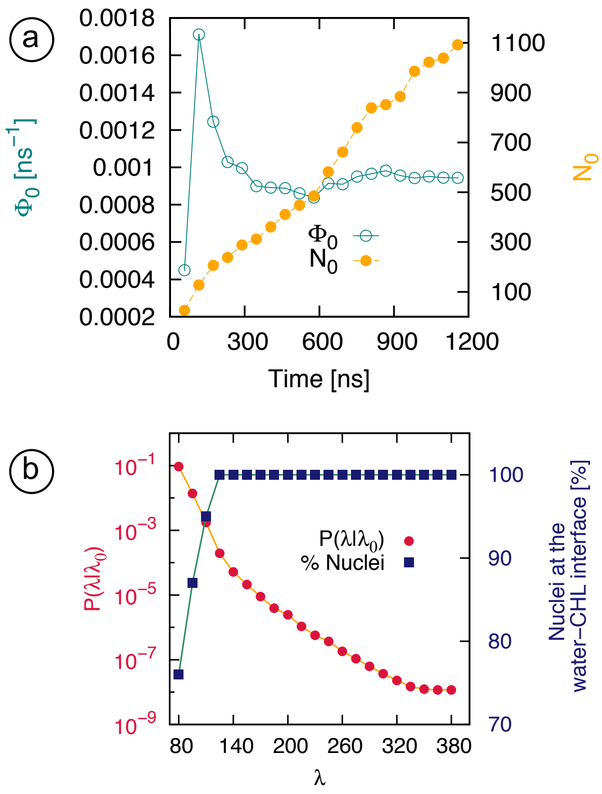

FIG. 2: a) Flux rate (Φ_0, left y-axis, empty circles) and number of direct crossing of the λ_0 interface (N_0, right y-axis, filled circles) as a function of simulation time. b) Calculated growth probability $P(\lambda|\lambda_0)$ and fraction of ice nuclei at the water-CHLM_{001}^{-OH} interface as a function of λ.

Finite Size Effects

Special care has to be taken when dealing with atomistic simulations of crystal nucleation from the liquid phase. Specifically, the presence of periodic boundary conditions can introduce significant finite effects, most notably spurious interactions between the crystalline nuclei and their periodic images. This artefact results in nonphysically large nucleation rates and/or artificially lower crystal growth speeds. In this work we have considered, due to the exceedingly computational cost of the FFS algorithm, simulation boxes with lateral dimensions of the order of 30 Å, which are not sufficient to avoid finite size effects throughout the whole of the nucleation process. Specifically, we measured the distance between the ice nuclei and their periodic images using the average set-set distance $d(A,B)$, which is defined as:

$$d(A,B) = \inf \lim_{x \in A, y \in B} |\mathbf{x} - \mathbf{y}| \tag{3}$$

where x and y are the position vectors of each oxygen atoms belonging to the largest ice nucleus (defined according to the order parameter λ) A and its first periodic image B respectively. At the FFS interface corresponding to λ=200, $d(A,B)$=4±2Å, which means that from that point onward, finite size effects are likely to play a role. This is why we could not extract in this case a quantitative measure for the nucleation rate - nor for the critical nucleus size.

CHARACTERISING THE ICE NUCLEI

Double-Diamond and Hexagonal Cages

Double-Diamond (DDC) and Hexagonal cages (HC) are the building blocks of cubic and hexagonal ice respectively. We have identified water molecules involved in DDC and/or HC within the largest ice nucleus in the system (defined according to the order parameter λ, see Eqs.1 and 2) following the topological criteria detailed in Ref. 15. The first step in order to locate DDC and HC is the construction of the ring network of the oxygen atoms belonging to each water molecule. In this work, we have obtained all the six-atom rings needed to build DDC and HC using King's shortest path criterion [21, 22] as implemented in the R.I.N.G.S. code [23]. The same distance cutoff of 3.2 Å used for the construction of the order parameter λ has been employed to determine the nearest neighbours of each oxygen atom. The same algorithm described in Ref. 15 has subsequently been used to determine DDC and HC.

Asphericity Parameter

Many different choices are available to quantify the asphericity of clusters of molecules. We have considered the gyration radius as well as the α (Δ in Ref. 24) and S asphericity parameters reported in Ref. 24. All of these quantities provided the same qualitative picture, so we have chosen to report the asphericity trends for α only, the latter being defined as:

$$\alpha = \frac{3}{2(tr\mathcal{T})^2} \sum_{i=1}^{3} (\mu_i - \bar{\mu})^2 \tag{4}$$

where μ_i are the three eigenvalues of the inertia tensor $\mathcal{T}$ for a given cluster, and $\bar{\mu} = \frac{tr\mathcal{T}}{3} = \frac{\sum_{i=1}^{3}(\mu_i)}{3}$

Spatial extent Δz

The spatial extent Δz for a given ice nucleus has been calculated as the difference between the minimum and maximum values of the z- components of the position vector of all the oxygens belonging to the nucleus. As the direction normal to the CHLM slab coincides to the z-axis of our simulation box, Δz provides a qualitative indication of the number of ice layers in the nuclei. Ice nuclei are defined to be on top of the CHLM surface ($Surf$, see main text) if the minimum value of the z-components of the position vector of all the oxygens belonging to the nucleus is < 18.0 Å, which correspond to the position of the main peak in the density profile of the water film along the z-axis. If this is not the case, the ice nuclei are considered to sit in the bulk of the water film ($Bulk$, see main text).

Nuclei at the CHLM-water Interface

Anisotropic Ice Nuclei on CHLM Crystals

Within the very early stages of the nucleation process, pre-critical nuclei (i.e. $\lambda <\sim 150$) are characterised by shapes very dissimilar to the hemispherical caps predicted by heterogeneous classical nucleation theory [25]. In particular, ice nuclei forming at the CHLM-water interface tend to be very elongated along a preferential direction, resulting in almost one-dimensional, chain-like nuclei. In Fig. S3 we show two representative configurations of such strongly anisotropic nuclei. The particular arrangement of the -OH groups on the (001) hydroxylated CHLM surface promotes the occurrence of one dimensional chains of water molecules, hydrogen bonded to the -OH groups, which stack upon each other to form the building blocks of the ice crystals (those cages of hydrogen bonded water molecules and -OH groups made of five- and six-membered rings described in the main text).

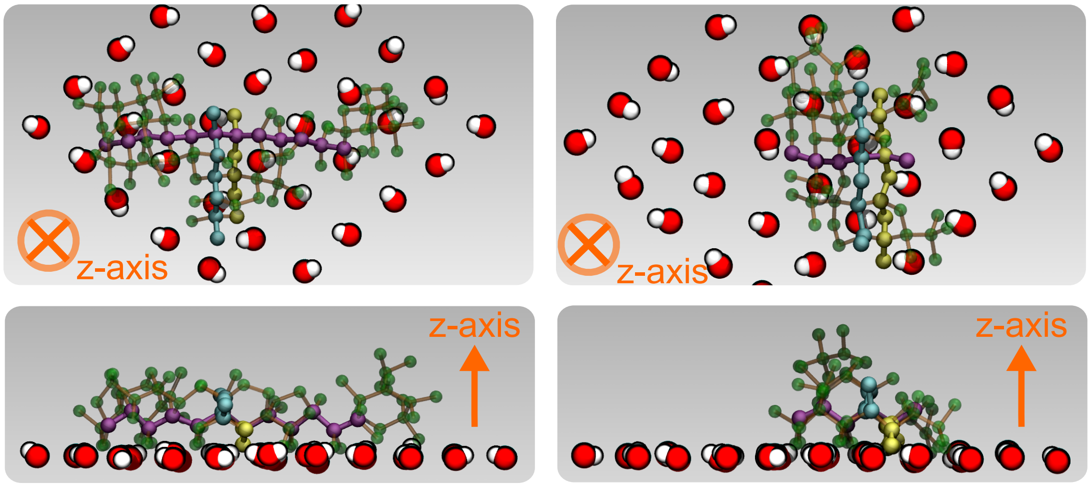

FIG. 3: Representative configurations of the strongly anisotropic, 1D chain-like nuclei observed within the early stages of ice nucleation on the hydrophilic (001) surface of CHLM crystals. Top and side view are shown for two different nuclei (left and right), where the z-axis runs along the normal to the water-CHLM interface. Only the hydroxyl groups of the CHL molecules within the simulation box are reported (red [oxygen] and white [hydrogen] spheres). Green (transparent) spheres correspond to the oxygen atoms of the water molecules belonging to the ice nuclei, while yellow, purple and cyan spheres correspond to selected 1D chains of said atoms which sit at different heights on the CHLM surface. Note that such structural motifs constitute the building blocks of I_c, and that these chains can be found along preferential orientations across the CHLM surface, due the particular arrangement of the hydroxyl groups.

Critical Nucleus Size

An estimate of the critical nucleus size N^C can be obtained directly from the crossing probabilities assuming that λ is a good reaction coordinate for the nucleation process [15]. In this scenario N^C is the value for which the committor probability $P^C(\lambda)$ for the nuclei to proceed towards the ice phase instead of shrinking into the liquid is equal to 0.5. Our forward flux sampling simulations indicate that $P^C(\lambda)$=0.5 corresponds in this case to a critical nucleus of 250 $\pm$ 40 water molecules. The estimate of the homogeneous critical nucleus size, obtained by means of the same approximate approach employed here, is N^C=500 $\pm$ 30 water molecules (as obtained by using the definition of λ employed in this work), more than two times larger than our estimate for the heterogeneous case. The value of N^C we have obtained for ice formation on CHLM crystals is comparable with that we have calculated in the case of ice formation on kaolinite [19]. This is consistent with the fact that the nucleation rates for ice formation on these two surfaces are basically the same (see main text). However, we must note that the presence of finite size effects prevent us to make a quantitative estimation of N^C in the case of ice formation of CHLM, so that this finding has to be interpreted as a qualitative measure of the ice nucleating ability of CHLM - which at the strong supercooling considered in here ($\sim$ 42 K) seems to be comparable to that of kaolinite.

FLEXIBILITY OF THE CHLM SLAB

The flexibility of the substrate, in this case of the CHLM crystalline slab, is a very important details when it comes to simulations of heterogeneous ice nucleation, both in the case of inorganic [1] and inorganic [26] impurities. In the case of CHLM crystals, we have chosen (as detailed above) to constrain the lateral dimension of the slab to the experimental values, and to constraint the very bottom of the CHL molecules, in order to mimic the effect of the underlying crystalline bulk. While we are confident this is the best option in terms of getting our simulations as close as possible to the experimental scenario, we cannot exclude that at the mild supercooling ($\sim$ 4 K) at which CHLM crystals are active as ice nucleating agents still, the crystalline slab could be characterised by a larger degree of flexibility. To assess this effect, we have studied the natural fluctuations of the water network (in exactly the same fashion as what we did for the initial stage of the FFS algorithm, that is by means of $\sim$ 1.2 μs long unbiased molecular dynamics simulations) for two additional configurations of the CHLM-water interface. In both cases we have considered a bilayer of CHL molecules (instead of a single CHLM crystalline slab) of which only the z-coordinate of the oxygen atoms of the bottom layer have been constrained. The upper CHLM slab (which is the one in contact with the water film) is entirely free to move. The two configurations differ as we have been sampling the NVT and NPT ensamble respectively, so in the latter case the later dimensions of the box were free to change along the way. In Fig. S4 we

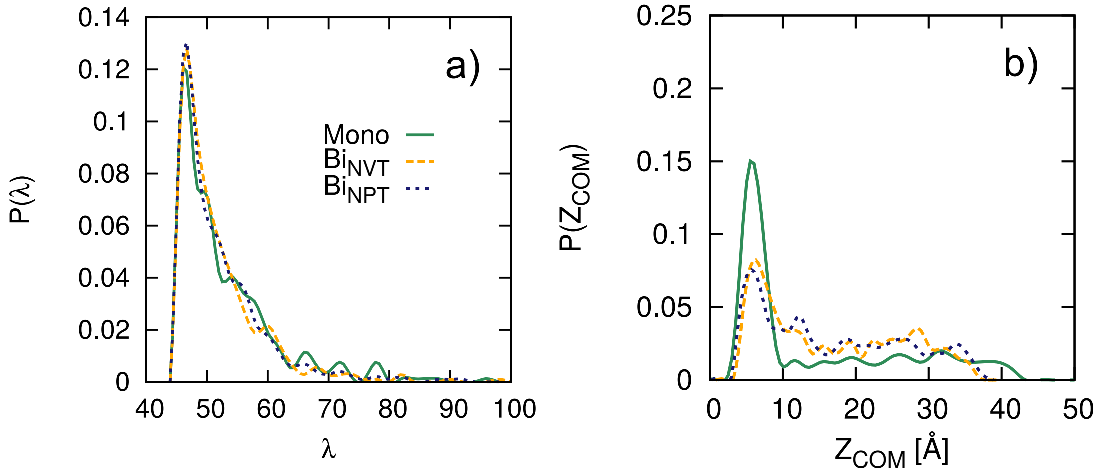

FIG. 4: Probability density distribution of a) the number λ of water molecules within the largest ice nuclei (containing at least 45 molecules) and b) the distance Z_{COM} of the center of mass of the same ice nuclei from the average z-coordinate of the -OH groups of the CHLM surface. The results for the single slab (Mono) investigated in the main text are compared with those obtained using a bilayer (of which only the oxygen atoms belonging to the CHL molecules within the lower slab are constrained, the upper slab in contact with the water is completely unconstrained) which has been simulated via sampling either the NVT (Bi_{NVT}) or the NPT (Bi_{NPT}) ensemble.

report the probability density distributions of the size of the ice nuclei and of the distance of their center of mass from the average z-coordinate of the -OH groups of the CHLM surface, for all the three configurations considered in here. Mono refers to the single CHLM slab discussed in the main text, while Bi_{NVT} and NPT (Bi_{NPT} stand for the results obtained from the bilayer configurations, sampling the NVT or NPT ensemble respectively. It can be seen that while the average dimension of the ice nuclei is basically the same for the three models, the probability of observing ice nuclei at the CHLM-interface is larger for the single CHLM slab than it is for the bilayer configurations. This implies that a larger degree of flexibility seems to hinder the ice templating effect of the surface, albeit whether or not the extent of pre-critical fluctuations can be directly related to the kinetics of ice formation remains yet to be seen.

ANHYDROUS CHOLESTEROL (CHLA)

Despite the fact that the monohydrate crystalline phase of cholesterol is the most relevant one in the context of both atmospheric science and cryobiology (as this is the polymorph that forms in aqueous environments), we did investigate the ice nucleating ability of the anhydrous phase (CHLA) as well. The latter has also been reported to be quite an efficient ice nucleating agent [27]. However, as opposite to CHLM which tend to crystallise in the planar plates described in the main text, CHLA tends to crystallise as needles, which we found rather difficult to characterise in terms of their ice nucleating ability. Why preliminary experimental results indicate that indeed CHLA possesses a strong ice nucleation ability at mild supercooling, a direct experimental comparison with CHLM is yet to be achieved. Having no indications about which particular crystalline face of CHLA is the one with the largest potential of nucleating ice, we have nonetheless investigated its only possibly hydrophilic surface (which similarly to CHLM can be obtained by cleavage along the bilayer structure, see e.g. Ref. 28). In contrast to what we have observed in the case of CHLM, we could not identify a clear templating effect of such surface, and indeed preliminary FFS simulations indicate that ice nuclei do not seem to favour the CHLA-interface (more then they tend to appear within the bulk of the water film). Thus, we can only hope that future experimental measurements will be able to identify the nucleation sites upon CHLM and CHLA as well. In fact, while CHLA may not be so relevant for practical applications, a direct comparison of its ice nucleating ability with CHLM could further our microscopic understanding of ice nucleation on organic crystals.

---

[1] G. C. Sosso, G. A. Tribello, A. Zen, P. Pedevilla, and A. Michaelides, The Journal of Chemical Physics **145**, 211927 (2016).

[2] P. Bjelkmar, P. Larsson, M. A. Cuendet, B. Hess, and E. Lindahl, J. Chem. Theory Comput. **6**, 459 (2010).

[3] J. B. Lim, B. Rogaski, and J. B. Klauda, J. Phys. Chem. B **116**, 203 (2012).
[4] J. L. F. Abascal, E. Sanz, R. G. Fernndez, and C. Vega, The Journal of Chemical Physics **122**, 234511 (2005).
[5] W. L. Jorgensen, J. Chandrasekhar, J. D. Madura, R. W. Impey, and M. L. Klein, The Journal of Chemical Physics **79**, 926 (1983).
[6] D. R. Nutt and J. C. Smith, J. Chem. Th. Comp. **3**, 1550 (2007).
[7] U. S. Midya and S. Bandyopadhyay, J. Phys. Chem. B **118**, 4743 (2014).
[8] B. M. Craven, Nature **260**, 727 (1976).
[9] E. Kkbenli, K. Sonkar, N. Sinha, and S. de Gironcoli, The Journal of Physical Chemistry A **116**, 3765 (2012).
[10] J. VandeVondele, M. Krack, F. Mohamed, M. Parrinello, T. Chassaing, and J. Hutter, Computer Physics Communications **167**, 103 (2005).
[11] M. Dion, H. Rydberg, E. Schrder, D. C. Langreth, and B. I. Lundqvist, Phys. Rev. Lett. **92**, 246401 (2004).
[12] J. Klime, D. R. Bowler, and A. Michaelides, J. Phys.: Condens. Matter **22**, 022201 (2010).
[13] S. Goedecker, M. Teter, and J. Hutter, Phys. Rev. B **54**, 1703 (1996).
[14] P. J. Steinhardt, D. R. Nelson, and M. Ronchetti, Physical Review B **28**, 784 (1983).
[15] A. Haji-Akbari and P. G. Debenedetti, Proceedings of the National Academy of Sciences , 201509267 (2015).
[16] G. A. Tribello, M. Bonomi, D. Branduardi, C. Camilloni, and G. Bussi, Computer Physics Communications **185**, 604 (2014).
[17] G. A. Tribello, F. Giberti, G. C. Sosso, M. Salvalaglio, and M. Parrinello, J. Chem. Theory Comput. **13**, 1317 (2017).
[18] Y. Bi and T. Li, The Journal of Physical Chemistry B **118**, 13324 (2014).
[19] G. C. Sosso, T. Li, D. Donadio, G. A. Tribello, and A. Michaelides, J. Phys. Chem. Lett. **7**, 2350 (2016).
[20] R. J. Allen, D. Frenkel, and P. R. t. Wolde, The Journal of Chemical Physics **124**, 194111 (2006).
[21] S. V. King, Nature **213**, 1112 (1967).
[22] D. S. Franzblau, Physical Review B **44**, 4925 (1991).
[23] S. Le Roux and P. Jund, Computational Materials Science **49**, 70 (2010).
[24] N. Rawat and P. Biswas, Physical Chemistry Chemical Physics **13**, 9632 (2011).
[25] V. Kalikmanov, *Nucleation Theory*, Lecture Notes in Physics, Vol. 860 (Springer Netherlands, Dordrecht, 2013).
[26] Y. Qiu, N. Odendahl, A. Hudait, R. Mason, A. K. Bertram, F. Paesani, P. J. DeMott, and V. Molinero, J. Am. Chem. Soc. **139**, 3052 (2017).
[27] R. B. Head, Nature **191**, 1058 (1961).
[28] H. S. Shieh, L. G. Hoard, and C. E. Nordman, Nature **267**, 287 (1977).
